# ß1-adrenergic blockers preserve neuromuscular function by inhibiting the production of extracellular traps during systemic inflammation in mice

**DOI:** 10.3389/fimmu.2023.1228374

**Published:** 2023-09-22

**Authors:** Camille H. Bourcier, Pauline Michel-Flutot, Laila Emam, Lucille Adam, Adeline Gasser, Stéphane Vinit, Arnaud Mansart

**Affiliations:** ^1^ END-ICAP, INSERM U1179, UVSQ-Université Paris-Saclay, Versailles, France; ^2^ Infection et Inflammation (2I), INSERM U1173, UVSQ-Université Paris-Saclay, Versailles, France

**Keywords:** inflammation, immune response, beta1-adrenergic blockers, extracellular traps, motor evoked potential, mouse model

## Abstract

Severe inflammation via innate immune system activation causes organ dysfunction. Among these, the central nervous system (CNS) is particularly affected by encephalopathies. These symptoms are associated with the activation of microglia and a potential infiltration of leukocytes. These immune cells have recently been discovered to have the ability to produce extracellular traps (ETs). While these components capture and destroy pathogens, deleterious effects occur such as reduced neuronal excitability correlated with excessive ETs production. In this study, the objectives were to determine (1) whether immune cells form ETs in the CNS during acute inflammation (2) whether ETs produce neuromuscular disorders and (3) whether an immunomodulatory treatment such as β1-adrenergic blockers limits these effects. We observed an infiltration of neutrophils in the CNS, an activation of microglia and a production of ETs following lipopolysaccharide (LPS) administration. Atenolol, a β1-adrenergic blocker, significantly decreased the production of ETs in both microglia and neutrophils. This treatment also preserved the gastrocnemius motoneuron excitability. Similar results were observed when the production of ETs was prevented by sivelestat, an inhibitor of ET formation. In conclusion, our results demonstrate that LPS administration increases neutrophils infiltration into the CNS, activates immune cells and produces ETs that directly impair neuromuscular function. Prevention of ETs formation by β1-adrenergic blockers partly restores this function and could be a good target in order to reduce adverse effects in severe inflammation such as sepsis but also in other motor related pathologies linked to ETs production.

## 1 Introduction

Inflammation is part of the innate defense mechanism of the body against infection. A dysregulated inflammation can become harmful and lead to multiple organ dysfunction. Among these, the central nervous system (CNS) is one of the first to be affected (1, 2). It displays neurotoxic processes characterized by electroencephalogram abnormalities, altered neurotransmission balance, increased excitotoxicity, impaired long-term potentiation, and neuronal apoptosis (3). Ultimately, severe inflammation leads to neurological dysfunctions (4) such as “intensive care unit-acquired weakness” (ICUAW), characterized by disorder of the neuromuscular system and a negative impact on physical function (5, 6). Many patients also develop a common complication characterized by an acutely altered mental state (from delirium to coma), a high mortality rate, and long-term cognitive impairments in surviving patients (7, 8).

These neurological dysfunctions result from CNS neuroinflammation. The generalized inflammation leads to the systemic production of pro-inflammatory cytokines that enter to the CNS and trigger glial cell activation, oxidative stress, and blood-brain barrier (BBB) permeabilization (9), resulting in peripheral immune cell infiltration (10, 11). The diverse neuroinflammatory processes then modulate neuronal activity, which contributes, at least in part, to the observed syndromes, such as reduced motoneuronal excitability in the case of ICUAW (12) and impaired somatosensory evoked potentials (2). However, the actors and pathways mediating this modulation of neuronal excitability remain to be explored.

Neuroinflammation involves diverse immune cells. The earliest recruited are microglia – resident macrophages (4, 13) and infiltrating neutrophils (14). Neutrophils are usually the first immune cells recruited following infection, and are the body’s first line of defense against bacterial infections. In CNS inflammation, they are recruited by activated astrocytes due to pro-inflammatory cytokines production (15). Neutrophils are mainly known for their innate immunity functions consisting of phagocytosis and the release of granules that contain toxic substances, allowing the elimination of extracellular microorganisms (16). However, in 2004, Brinkman et al. uncovered a new antimicrobial function of these neutrophils: extracellular trap (ET) formation (17). These neutrophil ETs (NETs) are mainly composed of chromatin, histones, and various proteins such as myeloperoxidase (MPO) and elastase. They help the organism eliminate pathogens such as bacteria (17). Dysregulation of NET formation can lead to deleterious outcomes such as vascular occlusions (18) and tissue damage (19, 20). Recently, this production of ETs by infiltrating neutrophils was shown to be involved in neuroinflammatory processes associated with CNS diseases and CNS traumatic injuries (21–23), especially in BBB disruption, spinal cord edema aggravation, and neuronal apoptosis (24). Interestingly microglia, which are the first level of defense against pathogens infection by making phagocytosis and antigen presentation in the CNS (25) have been shown to also produce extracellular traps (MiETs) *in vitro* (26–28) and *in vivo* (21, 28). However, data about this ET production in the CNS are lacking, and its potential involvement in neural dysfunction is yet to be explored.

In severe inflammation such as sepsis, drug development attempts have mainly focused on controlling inflammation or treating organ dysfunction, without significant benefit. Recently, interest has emerged in β1-adrenergic blockade due to the efficiency of β1-blockers in reducing inflammation and organ dysfunction. Clinical studies showed a reduction in patient mortality with β1-adrenergic blocker administration (29) by improving cardiovascular parameters such as heart rate (29, 30) or attenuating acute lung injury (31), probably through modulation of the immune response. Interestingly, β1-adrenergic blocker administration leads to a decrease in the production of pro-inflammatory cytokines such as TNF-α and IL-6 (31, 32). β1-adrenergic blocker also reduce the number of macrophages (33) and neutrophils (34, 35) involved in inflammatory and neuroinflammatory pathologies.

In this study, we hypothesized that (1) infiltrating neutrophils and activated microglia produce ETs in the CNS during severe inflammation, (2) these ETs are involved in the modulation of neuronal excitability and (3) based on their immunomodulatory properties, β1-blockers should reverse the adverse effect of ET formation on neuronal excitability. To address these important questions, we used a preclinical model of endotoxemic shock in mice. We evaluated motoneuronal excitability by recording gastrocnemius motor-evoked potentials, as well as ET production by neutrophils and microglia in the lumbar spinal cord where these gastrocnemius motoneurons are located.

## 2 Materials and methods

### 2.1 Ethics statement

These experiments were approved by the ethics committee of the University of Versailles Saint-Quentin-en-Yvelines (CEEA-47; APAFIS #38573-2022082610387745 v4) and complied with French and European laws regarding animal experimentation. The animals were housed in ventilated cages in a state-of-the-art animal care facility (2CARE animal facility, accreditation A78-322-3, France), with access to food and water ad libitum on a 12-hour light/dark cycle.

### 2.2 Experimental protocol

A total of 180 male Swiss mice (Janvier Labs, France, 25–37 g, 5–7 weeks old) were used in this study. A single intraperitoneal (i.p.) injection of LPS (22 mg/kg, from Escherichia coli O55:B5, L2880, Merck, Darmstadt, Germany) was used to create our preclinical endotoxemic shock model. Mice were divided into different groups: the LPS group (n = 49); the LPS + atenolol group: a selective β1-adrenergic blocker (atenolol, 0.1 mg/kg i.p, AstraZeneca, Courbevoie, France) was injected 6 hours post LPS injection (n = 33); and the LPS + sivelestat group: a selective ET inhibitor (sivelestat, ab14618, Abcam, Cambridge, UK) was injected 4 hours (10 mg/kg, i.p.), 8 hours (20 mg/kg, i.p.), and 12 hours (20 mg/kg, i.p.) post LPS injection (n = 16). Control groups were also used for each condition: the saline group (NaCl 0.9%, i.p.; n = 56), the atenolol group (atenolol, 0,1 mg/kg i.p., 6 hours post saline injection; n = 14), and the sivelestat group (NaCl 0.9%, i.p. + 3 sivelestat injections at 4, 8, and 12 hours post saline injection; n = 12) (**Supplemental Figure 1**).

Twenty-two hours post LPS injection, the mice were used for gastrocnemius MEP recordings or harvested for tissue analysis. This delay is sufficient to induce a severe inflammatory response but does not cause the death of the animals which occurs from the twenty-fourth hour.

### 2.3 Cell processing for flow cytometry analyses

The mice (n = 91) were first anesthetized with 5% isoflurane in 100% O_2_ and then maintained at 2.5% through a nose cone. The left carotid artery was cannulated. After euthanasia, the animals were perfused with ethylenediaminetetraacetic acid (EDTA, BP2482-1, Thermo Fisher Scientific, Waltham, MA, USA) in phosphate-buffered saline 1X (PBS, BP399-1, Thermo Fisher Scientific, Waltham, MA, USA) at 1 mL/g of body weight to remove blood from organs. The lumbar (L1–L6) spinal cord segment was then dissected out, cut into small pieces, and incubated in 2.5 mg/mL collagenase (11088858001, Sigma-Aldrich, Darmstadt, Germany) and RPMI solution (Roswell Park Memorial Institute, 1640 Gibco, 11875093, Thermo Fisher Scientific, Waltham, MA, USA) at 37°C for 20 minutes in 5% CO_2_. The tissue suspension was then passed through a 100-µm cell strainer (22-363-549, Fisher Scientific, Illkirch, France), resuspended in RPMI, and distributed in a 96-well plate (650 185, Greiner bio-one, Les Ulis, France) for staining.

To identify microglia, neutrophils, and ETs (saline: n = 16; LPS: n = 13; atenolol: n = 6; LPS + atenolol: n = 10; sivelestat: n = 6; LPS + sivelestat: n = 8), the cells were resuspended in a mix composed of cell viability marker (eBioscience™ Fixable Viability Dye eFluor™ 780, Thermo Fisher Scientific, Waltham, MA, USA) and SYTOX™ Green (Invitrogen- Thermo Fisher Scientific, Waltham, MA, USA) for 1 minute in PBS 1X. Cells were then incubated with anti-TFAM antibody (20 minutes in RPMI at 4°C), followed by secondary antibody (20 minutes in RPMI at 4°C, see **Table 1** for antibody details) and an antibody mix (30 minutes at 4°C, **Table 1**). H3 antibody was conjugated with an antibody conjugation kit (DyLight 594, Abcam, ab201801, Paris, France) according to the manufacturer’s instructions.

**Table 1:** Antibodies used for cell processing before flow cytometry analyses.

Target	Reference	Fluorochrome
CD45	BioLegend Cat# 103137, RRID : AB_2561392	BV510
Ly6C	BioLegend Cat# 128012, RRID : AB_1659241	PerCP/Cy5.5
Ly6G	BioLegend Cat# 127618, RRID : AB_1877261	PE-Cy7
IA/IE	BioLegend Cat# 107622, RRID : AB_493727	AlexaFluor700
CD11b	BioLegend Cat# 101224, RRID : AB_755986	Pacific blue
TFAM Secondary antibody goat anti rabbit	Boster (PB9447) (Abcam Cat# ab130805, RRID : AB_11156672)	Unconjugated APC
MPO	Hycult Biotech Cat# HM1051, RRID : AB_533151	PE
H3 (citrulline R2 + R8 +R17)	Abcam Cat# ab5103, RRID : AB_304752	DyLight 594

For the evaluation of β1-adrenergic receptor quantity (saline: n = 11 mice; LPS: n = 12; LPS + atenolol: n = 9), cells were incubated with the antibody mix shown in **Table 2** for 30 minutes at 4°C in PBS 1X.

**Table 2:** Antibodies used for β1-adrenergic receptors study before flow cytometry analysis.

Target	Reference	Fluorochrome
CD45	BioLegend Cat# 103137, RRID : AB_2561392	BV510
Ly6C	BioLegend Cat# 128012, RRID : AB_1659241	PerCP/Cy5.5
Ly6G	BioLegend Cat# 127618, RRID : AB_1877261	PE-Cy7
IA/IE	BioLegend Cat# 107622, RRID : AB_493727	AlexaFluor700
CD11b	BioLegend Cat# 101224, RRID : AB_755986	Pacific blue
ADRB1	Bioss Cat# bs-0498R-PE, RRID : AB_11110613	PE

The data was collected with a flow cytometer BD LSRFortessa™ III (BD Life Sciences, Franklin Lakes, NJ, USA) and analyzed using FlowJo™ v10.8.1 Software (RRID : SCR_008520, BD Life Sciences, Franklin Lakes, NJ, USA).

### 2.4 Immunohistochemistry and tissue processing

The animals used for immunofluorescence analysis (saline: n = 6 mice; LPS: n = 6, LPS + atenolol: n=6) were anesthetized using 5% isoflurane in 100% O_2_ for induction and maintained at 2.5% through a nose cone. The mice were then euthanized by intracardiac perfusion of heparinized 0.1% (Panpharma, Luitré-Dompierre, France) saline solution (NaCl 0.9%). The lumbar segments (L1–L6) of the spinal cord were collected, fixed for 24 hours at 4°C (formalin 10%, HT501128 Sigma-Aldrich, Darmstadt, Germany), and cryoprotected for 48 hours in 30% sucrose in PBS 1X (Pharmagrade, 141621, AppliChem, Darmstadt, Germany), then frozen and stored at -80°C. Transversal sections of 30 µm were obtained with a cryostat (NX70, Thermo Fisher Scientific, Waltham, MA, USA) and stored at -20°C in cryoprotectant (300 g sucrose, 300 mL ethylene glycol [BP230‐4, Fisher Scientific, Illkirch, France], and 10 g polyvinylpyrrolidone 40 [PVP40‐100G, Sigma‐Aldrich, Darmstadt, Germany]), adjusted at 1 L with PBS 1X. After washing with PBS 1X, antigen retrieval (2.94 g/L tris-sodium citrate solution at pH 6) was realized for 20 minutes at 96°C. The sections were then permeabilized (0.5% triton X-100 [X100-100ML, Sigma-Aldrich, Darmstadt, Germany]) in TBS 1X, blocked (30 minutes at room temperature) in a saturation solution (4% BSA [Bovine Serum Albumin, A0336-50ML, Sigma-Aldrich, Darmstadt, Germany], 4% NDS [Normal Donkey Serum, 566460, Sigma-Aldrich, Darmstadt, Germany], 0.05% triton X-100, 0.05% Tween 20 [BP337-100, Fisher Scientific, Illkirch, France]) in TBS 1X, and incubated overnight at room temperature with primary antibodies (**Table 3**). After washing, secondary antibodies (**Table 3**) were incubated for 2 hours at room temperature in saturation solution. Nuclei were labeled with DAPI (D1306, Thermo Fisher Scientific, Waltham, MA, USA) for 10 minutes at room temperature (0.0025 mg/mL in saturation solution) and then washed again. The sections were mounted on slides, dried, and cover-slipped with fluorescent mounting medium (fluoromount G, Invitrogen-Thermo Fisher Scientific, Waltham, MA, USA). Images of the different sections were captured with a Hamamatsu ORCA-R2 camera mounted on an Olympus IX83 P2ZF microscope (Tokyo, Japan) or with a confocal microscope (Leica TCS SP8 microscope, Wetzlar, Germany) for higher magnification. Pictures were analyzed using ImageJ 1.53n software (ImageJ, RRID : SCR_003070, National Institutes of Health, Bethesda, MD, USA).

**Table 3:** Antibodies used for immunofluorescence analyses.

Target	Reference	Dilution
Ly6G (Rat)	BioLegend Cat# 127601, RRID : AB_1089179	1/100
H3 (citrulline R2 + R8 + R17) (Rabbit)	Abcam Cat# ab5103, RRID : AB_304752	1/100
MPO (Rabbit)	Thermo Fisher Scientific Cat# PA5-16672, RRID : AB_11006367	1/100
Iba1 (goat)	Abcam Cat# ab5076, RRID : AB_2224402	1/400
Donkey anti-Rat, Alexa Fluor™ 488	Thermo Fisher Scientific Cat# A-21208, RRID : AB_2535794	1/2000
Donkey anti-Rabbit, Alexa Fluor™ 594	Thermo Fisher Scientific Cat# A-21207, RRID : AB_141637	1/2000
Donkey anti-Goat, Alexa Fluor™ 647	Thermo Fisher Scientific Cat# A-21447, RRID : AB_2535864	1/2000

### 2.5 Electrophysiological recordings of gastrocnemius motor evoked potentials

The excitability of motoneurons associated with the gastrocnemius muscle was evaluated (saline: n = 15 mice; LPS: n = 10; atenolol: n = 8; LPS + atenolol: n = 8; sivelestat: n = 6; LPS + sivelestat: n = 8) by electrophysiological recordings of the gastrocnemius (MEP). Briefly, anesthesia was induced using isoflurane (5% in 100% O_2_) and maintained (1%) through a nose cone. Two microneedle electrodes were inserted in the left and right gastrocnemius. A third electrode was placed subcutaneously as a ground electrode. The electrodes were left in place for the entire duration of the experiment.

Trans-spinal magnetic stimulation, applied above CNS structures (spinal cord), is known to induce recordable MEP (36). This stimulation was performed using a magnetic stimulator MAGPRO R30 (Magventure, Farum, Denmark). The device is connected to a figure-of-eight coil (Cool-B65, Magventure, Farum, Denmark) that delivers a unique biphasic pulse with a stimulus intensity expressed as a percentage of the maximum output of the machine (%MO). Each animal was placed on a non-magnetic 3D-printed custom-made stereotaxic apparatus on the figure-of-eight coil, orientated at 90° to obtain the maximum amplitude (37), with the center of the coil under the cervical level of the animal. Each animal received five magnetic stimulation (MS) pulses, from 40% to 100% MO, with 10% increases (35 pulses in total), with an interpulse duration above 15 seconds to avoid repetitive, low-frequency MS-like effects. Gastrocnemius MEPs were amplified (gain, 1 k; A-M Systems, Everett, WA, USA) and band pass-filtered (100 Hz to 10 kHz). The signals were then digitized with an eight-channel Powerlab data acquisition device (acquisition rate: 100 k/s; AD Instruments, Dunedin, New Zealand) connected to a computer and analyzed using LabChart 8 Pro software (RRID : SCR_017551, AD Instruments, Dunedin, New Zealand).

### 2.6 Blood-spinal cord barrier permeability

BSCB permeability was determined using Evans blue (EB) (E2129, Sigma-Aldrich, Darmstadt, Germany) extravasation. The mice (saline: n = 8; LPS: n = 8) were anesthetized using 5% isoflurane in 100% O_2_ for induction and maintained at 2.5% through a nose cone. A solution of 50 mg/mL of EB in saline solution was administered intravenously by a retro-orbital route (0.1 mL/10 g body weight). After 2.5 hours, the mice were anesthetized as described previously and transcardially perfused with heparinized 0.9% NaCl after euthanasia. The spinal cord was then collected. Each spinal cord was lysed in 200 µL of a 7% perchloric acid solution (311421-50ML, Sigma-Aldrich, Darmstadt, Germany) with five Zirconium-Silicate spheres (6913500, MP Biomedicals, Illkirch, France) using a Precellys Evolution homogenizer (P000062-PEVO0-A, Bertin Technologies) and then centrifuged at 10,000 G for 30 minutes. The samples were diluted three times in ethanol, and the fluorescence intensity was measured with an Odyssey CLx Infrared Imaging System (LI-COR Biosciences). The concentration of dye/g of tissue was determined using a standard curve of EB in ethanol 100%.

To measure section fluorescence, fluorescein isothiocyanate (FITC)-albumin (A9771,Sigma-Aldrich, Darmstadt, Germany, dissolved at 20mg/mL in saline solution) was intravenously administered by a retro-orbital route (0,1 mL). The circulation period of FITC-albumin was ensured for 30 min prior to euthanasia. The mice were then anesthetized as described previously and euthanized. The spinal cord was then collected and Snap-freeze immediately in liquid nitrogen cooled isopentane. Sections of 30 µm were sliced, then mounted on slides, dried, cover-slipped with fluorescent mounting medium (fluoromount G, Invitrogen-Thermo Fisher Scientific, Waltham, MA, USA). Images were captured with a Hamamatsu ORCA-R2 camera mounted on an Olympus IX83 P2ZF microscope (Tokyo, Japan).

### 2.7 Data processing and statistical analysis

For gastrocnemius MEP analysis, five MEPs for each side of every animal were superimposed and averaged, and the amplitude and latency were quantified with LabChart 8 Pro software (RRID : SCR_017551, AD Instruments, Dunedin, New Zealand). Two-way ANOVA was used to compare each condition obtained with the same MO (percentage of stimulation for MEP measure from 40% to 100% MO) and animal groups.

For flow cytometry analysis, FlowJo v10.8.1 Software (RRID : SCR_008520, BD Life Sciences, Franklin Lakes, NJ, USA) was used to study cell populations and quantification of mean fluorescence intensity (MFI) for ET markers and β1-adrenergic receptor expression.

For immunohistochemistry analysis, ImageJ 1.53n software (ImageJ, RRID : SCR_003070, National Institutes of Health, Bethesda, MD, USA) was used to evaluate Iba1 labeling on the entire spinal cord section. A minimum of 5 spinal sections per animal were used to evaluate the MFI and the percentage of surface occupied by Iba1 labeling per slide for each group.

All data are presented as mean ± SD. Statistics were considered significant when p<0.05 (*p<0.05, **p<0.01, ***p<0.001). Normality was tested with the Shapiro–Wilk test. The difference between two groups was assessed with the two-tailed unpaired Student’s t-test or the non-parametric Mann–Whitney test. Between at least three groups, the difference was assessed by one-way ANOVA, followed by Fisher’s LSD *post hoc* or the Kruskal–Wallis test followed by Dunn’s test if normality failed. SigmaPlot 12.5 software (RRID : SCR_003210 Systat Software, San Jose, CA, USA) was used for all analyses.

## 3 Results

### 3.1 LPS injection induced microglia activation and an increase in neutrophil infiltration in the spinal cord.

**Figure 1:**
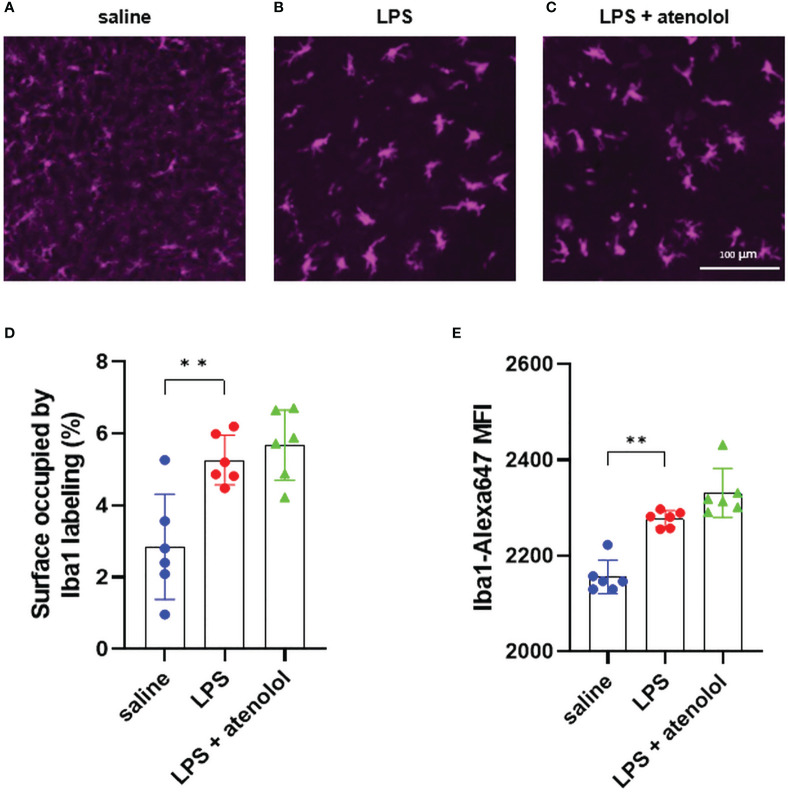
Atenolol did not change activation state of microglia. Representative pictures of ventro-lateral quadrant of the grey matter of spinal cord transversal sections (low thoracic to high lumbar segment), labeled with Iba1, for saline **(A)**, LPS **(B)**, and LPS + atenolol **(C)** groups. Quantification of surface occupied by Iba1 labeling per spinal cord section **(D)** and mean fluorescence intensity (MFI) of Iba1 labeling **(E)** for saline (blue), LPS (red) and LPS + atenolol (green) groups. Saline n=6, LPS n=6, LPS + atenolol n=6. ** p<0.01.

We first evaluated microglia activation and the presence of neutrophils in the spinal cord 22-hours after lipopolysaccharide (LPS) injection, using immunohistochemistry and flow cytometry. Microglia were identified as CD11b^+ and^ CD45^int^ cells (**Supplemental Figure 2**). No significant change was observed between all groups in the frequency of microglia among living cells in the spinal cord (**Supplemental Figure 3**). Activation of these microglia was then identified by change morphology characterized by swelling of the microglial cell body, thickening of the proximal processes, and reduction of the distal branching (38), identifiable by immunolabeling microglia with Iba1 on spinal cord section. LPS injection induced morphological changes of microglia showing microglia activation (**Figure 1B**) compared to the saline group (**Figure 1A**), with a significant increase in surface occupied by Iba1 staining (**Figure 1D**) and in mean fluorescence intensity of Iba1 staining (**Figure 1E**) for the LPS group compared to the saline group. The administration of atenolol in animals injected with LPS (LPS + atenolol group) did not affect the morphology of activated microglia (**Figures 1C–E**).

Neutrophils were identified as CD11b^+^, CD45^high^, and Ly6G^+^ cells (**Supplemental Figure 2**). The frequency of neutrophils in the CNS among live cells was increased in the spinal cord 22 hours after LPS injection compared to the saline group (1.19 ± 0.72% *vs.* 0.25 ± 0.17%, respectively, p<0.001), whereas the frequency of neutrophils was similar between the LPS group and LPS + atenolol group (**Supplemental Figure 3**). We confirmed the presence of infiltrated neutrophils in the spinal cords of LPS-treated animals by immunostaining of Ly6G on spinal cord sections (**Supplemental Figure 3**). This effect of LPS on neutrophils was correlated with an increase of Evans blue (EB) leakage into the spinal cord tissue (saline group: 27.31 ± 7.05 *vs*. LPS group: 81.34 ± 27.08 µg/g of spinal cord, p<0.001), (**Supplemental Figure 4**).

### 3.2 LPS increased the number of β1- adrenergic receptors on neutrophils and microglia

We observed the presence of β1-adrenergic receptors on the surfaces of neutrophils (**Supplemental Figures 5D, E**) and microglia (**Supplemental Figure 5F**). The administration of LPS induced an increase in the expression of these receptors in blood neutrophils (1,521 ± 1,201 *vs.* 499 ± 604.5 [mean fluorescence intensity, MFI] respectively, p<0.001) (**Supplemental Figures 5A, D**) and microglia (14,764 ± 2,135 *vs.* 1,370 ± 767.9 (MFI) respectively, p<0.001) (**Supplemental Figure 5C** and **4F**) compared to the saline group. The administration of atenolol did not alter the expression of β1-adrenergic receptors in neutrophils (**Supplemental Figures 5D, E**) and microglia (**Supplemental Figure 4F**) in the LPS + atenolol group or atenolol group.

### 3.3 Atenolol decreased spinal ET production in endotoxemic mice

To determine whether these infiltrating neutrophils and resident microglia could form ETs in the spinal cord following LPS injection, we studied the expression of four individual markers of ETs: citrullinated histone H3 (H3 citr) involved in chromatin decondensation, myeloperoxidase (MPO, a granular enzyme), SYTOX (a DNA intercalant agent), and TFAM (a protein linked to mitochondrial DNA) (39, 40). Studying ETs with a combination of these markers allows for being more specific and avoiding false positives.

**Figure 2:**
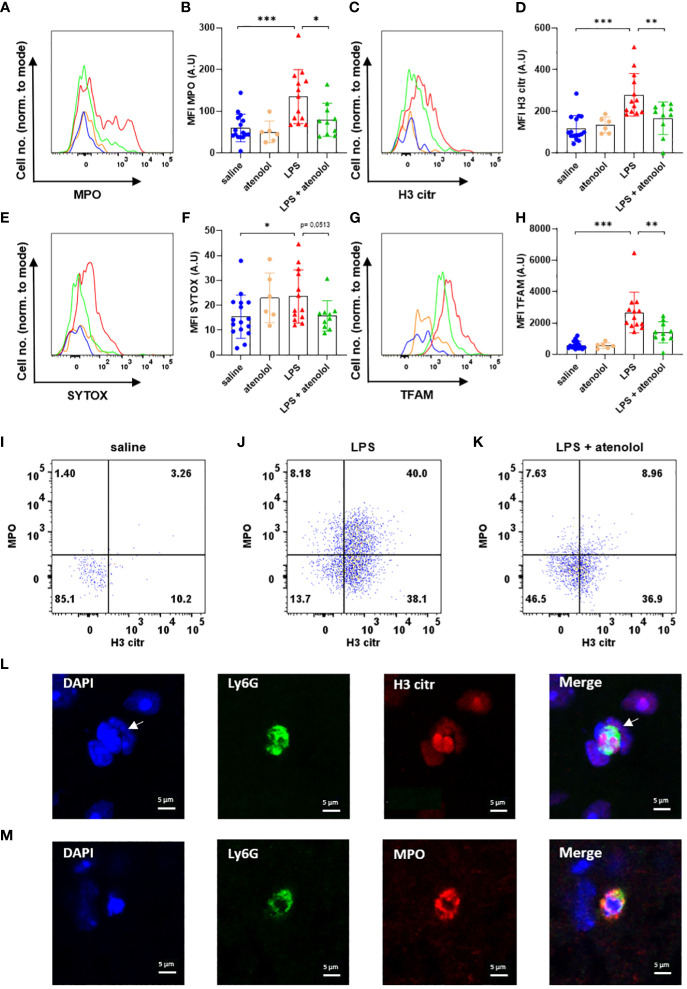
Atenolol decreased the production of NETs induced by LPS. Fluorescence intensity for a representative animal of each group for the extracellular trap markers MPO **(A)**, H3 citr **(C)**, Sytox **(E)** and TFAM **(G)** in saline (blue), atenolol (orange), LPS (red) and LPS + atenolol (green). Quantification of mean fluorescence intensities (MFIs) in live neutrophils for the extracellular trap markers MPO **(B)**, H3 citr **(D)**, Sytox **(F)** and TFAM **(H)** for saline (blue), atenolol (orange), LPS (red) and LPS + atenolol (green) groups. Representative dot plot of living neutrophils co-expressing MPO and H3 citr in saline **(I)**, LPS **(J)** and LPS + atenolol **(K)** groups. Representative pictures of Ly6G+ neutrophils (green) labeling with citrullinated histone H3 (H3 citr) (red). The nucleus of cells was labeled with DAPI (in blue). Arrow shows a neutrophil producing an ET **(L)**. Representative pictures of Ly6G+ neutrophils (green) labeling with myeloperoxidase (MPO) (red). The nucleus of cells was labeled with DAPI (in blue) **(M)**. Saline n=16, atenolol n=6, LPS n=13, LPS + atenolol n=10. * p<0.05, ** p<0.01, *** p<0.001.

**Figure 3:**
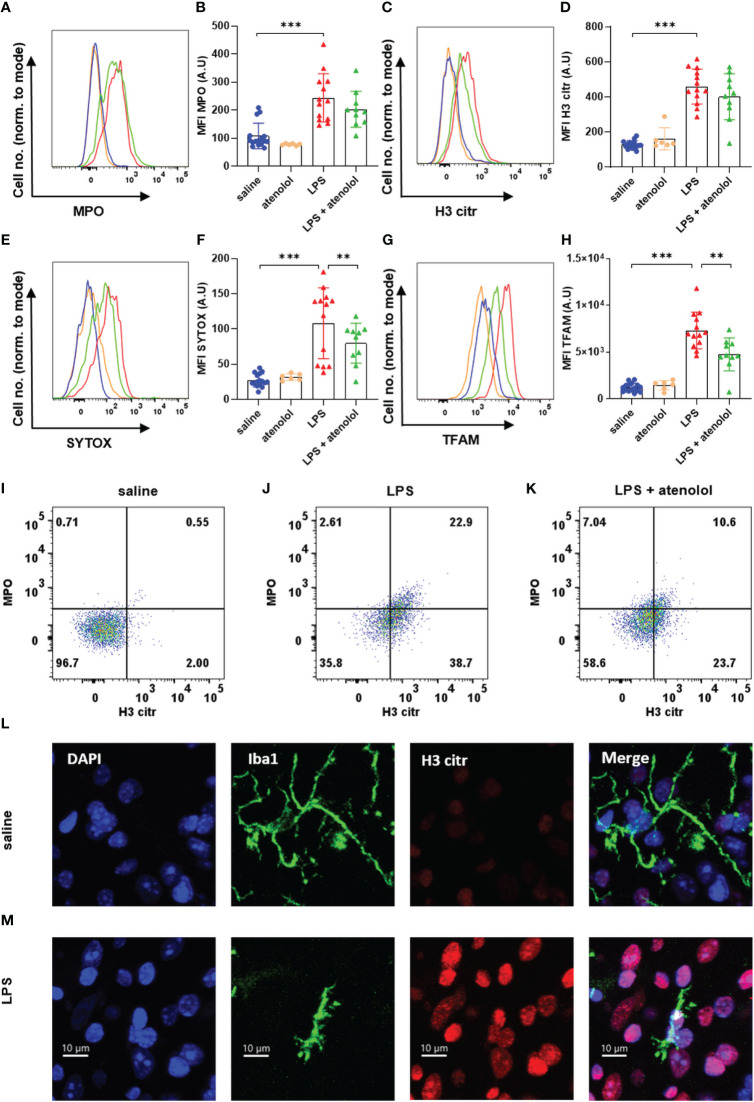
Atenolol decreased the production of MiETs induced by LPS. Fluorescence intensity for a representative animal of each group for the extracellular trap markers MPO **(A)**, H3 citr **(C)**, Sytox **(E)** and TFAM **(G)** in saline (blue), atenolol (orange), LPS (red) and LPS + atenolol (green) groups. Quantification of mean fluorescence intensities (MFIs) in live microglia for the extracellular traps markers MPO **(B)**, H3 citr **(D)**, Sytox **(F)** and TFAM **(H)** for saline (blue), atenolol (orange), LPS (red) and LPS + atenolol (green) groups. Representative dot plot of living microglia co-expressing MPO and H3 citr in saline **(I)**, LPS **(J)** and LPS + atenolol **(K)** groups. Representative pictures of Iba1 + microglia (green) labeling with citrullinated histone H3 (H3 citr) (red). The nucleus of cells was labeled with DAPI (in blue) for saline **(L)** and LPS **(M)** groups. Saline n=16, atenolol n=6, LPS n=13, LPS + atenolol n=10. ** p<0.01, *** p<0.001.

Expressions of MPO in neutrophils (**Figures 2A, B**) and microglia (**Figures 3A, B**), H3 citr in neutrophils (**Figures 2C, D**) and microglia (**Figures 3C, D**), SYTOX in neutrophils (**Figures 2E, F**) and microglia (**Figures 3E, F**), and TFAM in neutrophils (**Figures 2G, H**) and microglia (**Figures 3G, H**) were significantly increased 22 hours following the injection of LPS compared to saline group. The administration of atenolol in animals injected with LPS (LPS + atenolol group) led to a reduction in MPO (**Figures 2A, B**), H3 citr (**Figures 2C, D**), and TFAM (**Figures 2G, H**) expression in neutrophils compared to the LPS group, whereas only a tendency towards decrease could be observed for SYTOX expression (**Figures 2E, F**). In microglia, atenolol administration in animals treated with LPS (LPS + atenolol group) led to a reduction of the expression in SYTOX (**Figures 3E, F**) and TFAM (**Figures 3G, H**) and tended to reduce the expression of MPO (**Figures 3A, B**) and H3 citr (**Figures 3C, D**) compared to the LPS group. As expected, the administration of atenolol alone (atenolol group) had no significant effect on the expression of MPO in neutrophils (**Figure 2B**) and microglia (**Figure 3B**), H3 citr in neutrophils (**Figure 2D**) and microglia (**Figure 3D**), SYTOX in neutrophils (**Figure 2F**) and microglia (**Figure 3F**), and TFAM in neutrophils (**Figure 2H**) and microglia (**Figure 3H**) compared to the saline group.

We also evaluated the frequency of neutrophils and microglia co-expressing H3 citr and MPO, which is a better proxy for ET formation. An increased expression of MPO alone, for instance, could reflect cell degranulation. We found an increase in the co-expression of H3 citr and MPO following LPS injection in both neutrophils and microglia (**Figures 2J** and **3J** respectively) compared to the saline group (respectively **Figures 2I** and **3I**). The administration of atenolol in LPS-treated animals (LPS + atenolol group) reduced the proportion of neutrophils (**Figure 2K**) or microglia (**Figure 3K**) positive for MPO and H3 citr co-labeling compared to the LPS group (respectively **Figures 2J** and **3J**).

To deepen our analysis, we also identified ET formation in neutrophils and microglia on fixed spinal cord tissue. We visualized the intraspinal NET production using a neutrophil-specific marker (Ly6G) combined with a marker of ETs (H3 citr) in LPS-treated animals. Among the neutrophils (Ly6G^+^ cells), a H3 citr labeling was observed outside the cell contour (white arrow) (**Figure 2L**) as well as a MPO labeling (**Figure 2M**). A similar observation was performed for microglia (identified as Iba1^+^ cells) for H3 citr expression in the LPS and saline group (**Figures 3L, M**).

### 3.4 Atenolol preserved motoneuronal excitability in endotoxemic mice

**Figure 4:**
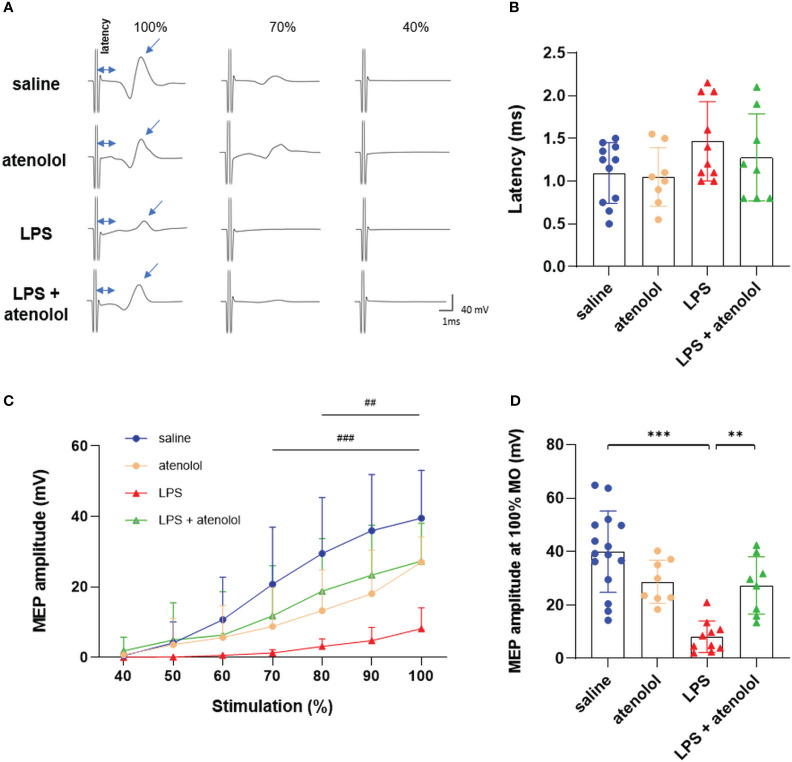
Atenolol limited the LPS-induced decrease in MEP amplitude. Representative motor-evoked potential (MEP) recording at 100%, 70%, and 40% of the maximum capacity of the TMS coil **(A)**. Latency (time between stimulation and recorded MEP) for saline (blue), atenolol (orange), LPS (red) and LPS+ atenolol (green) **(B)** groups. Quantification of MEP amplitude after stimulation from 40% to 100% of the coil’s maximum for saline (blue), atenolol (orange), LPS (red) and LPS+ atenolol (green) **(C)** groups. Individual values for stimulation at 100% of the coil’s capacity for saline (blue), atenolol (orange), LPS (red) and LPS+ atenolol (green) **(D)**. Saline n=15, atenolol n=8, LPS n=10, LPS + atenolol n=8. ** p<0.01, *** p<0.001. ^###^ p<0.001 saline *vs* LPS group from 70 to 100%. ^##^ p<0.01 LPS *vs* LPS + atenolol group from 80 to 100%.

β1-adrenergic blockers have a negative effect on the production of ETs. We wondered whether this antagonist could also have an impact on the motoneuronal excitability during severe inflammation. To address this question, gastrocnemius motor-evoked potentials were assessed in all groups (**Figure 4**). No significant difference was found in terms of latency (i.e., the delay between the stimulation artifact and the beginning of the motor-evoked potential, MEP), which represents the conduction velocity (**Figure 4B**). However, the administration of LPS reduced the gastrocnemius MEP amplitude 22 hours after the injection. This reduction was significant from 70% to 100% maximum output (MO) for the LPS group compared to saline-injected animals (8.15 ± 5.89 *vs*. 39.93 ± 15.16 mV, respectively, p<0.001). Interestingly, atenolol injection in LPS animals preserved gastrocnemius MEP amplitude (ranging from 80% to 100% MO) compared to LPS alone (**Figures 4A, C, D**). The administration of atenolol alone (atenolol group) had no significant effect on gastrocnemius MEP amplitude compared to the saline group (**Figures 4C, D**).

### 3.5 Sivelestat decreased spinal ET production in endotoxemic mice

β1-adrenergic blockers have overall anti-inflammatory effects. We wondered whether the decrease in gastrocnemius MEP amplitude we observed could be related to the increase in ET production by neutrophils and microglia after atenolol administration. To test this hypothesis, we used sivelestat, an inhibitor of the neutrophil elastase involved in the formation of ETs (41, 42).

**Figure 5:**
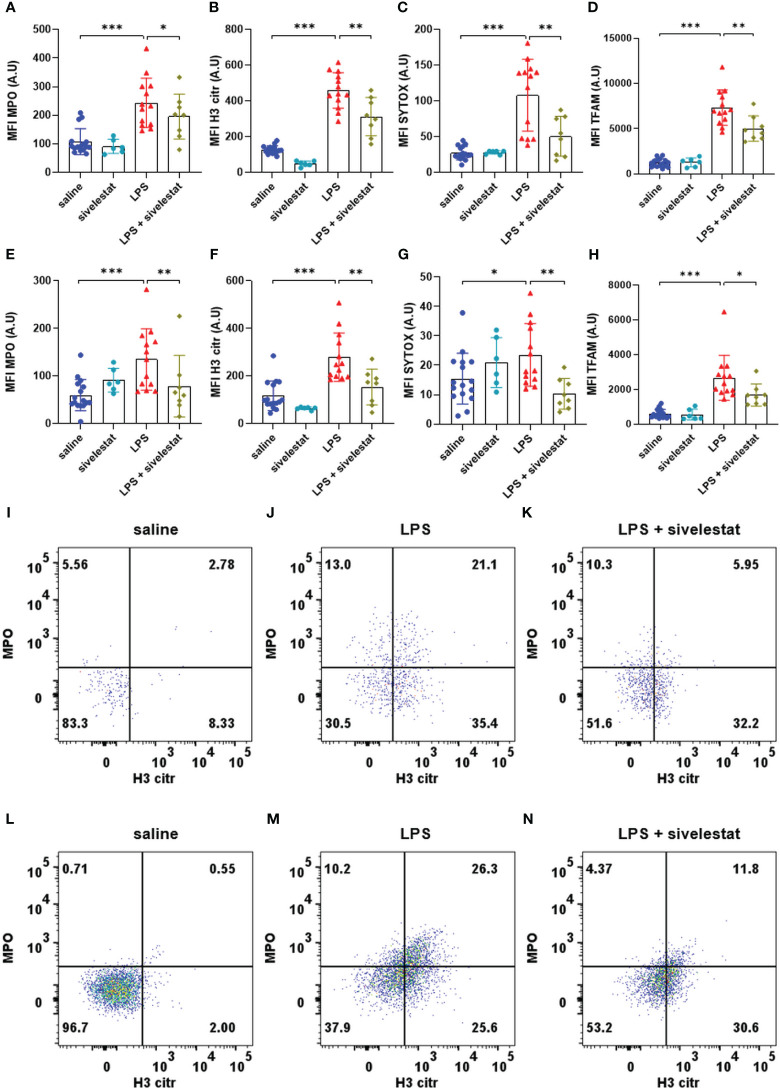
Sivelestat decreased the production of ETs induced by LPS. Quantification of mean fluorescence intensities (MFIs) of extracellular trap markers MPO **(A)**, H3 citr **(B)**, Sytox **(C)** and TFAM **(D)** for saline (blue), sivelestat (turquoise), LPS (red) and LPS + sivelestat (khaki) groups in live neutrophils. Quantification of MFI of extracellular traps markers MPO **(E)**, H3 citr **(F)**, Sytox **(G)** and TFAM **(H)** for saline (blue), sivelestat (turquoise), LPS (red) and LPS + sivelestat (khaki) groups in live microglia. Representative dot plot of live neutrophils co-expressing MPO and H3 citr in saline **(I)**, LPS **(J)** and LPS + sivelestat **(K)**. Representative dot plot of living microglia co-expressing MPO and H3 citr in saline **(L)**, LPS **(M)** and LPS + sivelestat **(N)** groups. Saline n=16, sivelestat n=6, LPS n=13, LPS + sivelestat n=8. * p<0.05, ** p<0.01, ***p<0.001.

We first verified the effect of sivelestat on NET and MiET production in our preclinical model of severe inflammation. As expected, the injection of sivelestat in LPS animals (LPS + sivelestat group) significantly reduced MPO (**Figure 5A**), H3 citr (**Figure 5B**), SYTOX, (**Figure 5C**) and TFAM (**Figure 5D**) expression in neutrophils compared to the saline-injected LPS group. Similar results were observed in microglia with sivelestat injection in LPS animals, i.e., a significant reduction in MPO (**Figure 5E**), H3 citr (**Figure 5F**), SYTOX, (**Figure 5G**) and TFAM (**Figure 5H**) expression compared to the saline-injected LPS group. In the same way, the injection of sivelestat in LPS-treated animals (LPS + sivelestat group) reduced the proportion of neutrophils (**Figure 5K**) and microglia (**Figure 5N**) positive cells for the combined MPO and H3 citr markers compared to the LPS group (respectively **Figures 5J, M**).

The administration of sivelestat alone (sivelestat group) had no significant effects on the expression of all the ET markers in neutrophils (respectively **Figures 5A–D**) and microglia (respectively **Figures 5E–H**) compared to the saline group.

### 3.6 ET formation was directly involved in motoneuronal excitability

**Figure 6:**
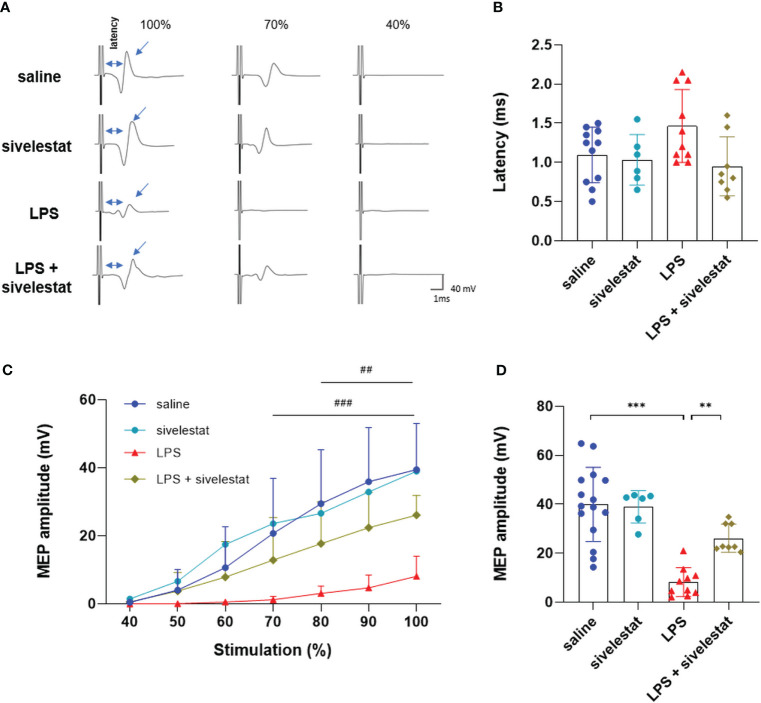
Sivelestat limited the LPS-induced decrease MEP amplitude. Representative motor evoked potential recording at 100%, 70%, and 40% of the maximum capacity of the TMS coil **(A)**. Latency (time between stimulation and recorded MEP) for saline (blue), sivelestat (turquoise), LPS (red) and LPS+ sivelestat (khaki) **(B)** groups. Quantification of amplitude after stimulation from 40% to 100% of the coil’s maximum for saline (blue), sivelestat (turquoise), LPS (red) and LPS+ sivelestat (khaki) **(C)** groups. Individual values for stimulation at 100% of the coil’s maximum capacity for saline (blue), sivelestat (turquoise), LPS (red) and LPS+ sivelestat (khaki) **(D)** groups. Saline n=15, sivelestat n=6, LPS n=10, LPS + sivelestat n=8. ** p<0.01, *** p<0.001. ^###^ p<0.001 saline *vs* LPS group from 70 to 100%. ^##^ p<0.01 LPS *vs* LPS + sivelestat group from 80 to 100%.

Gastrocnemius MEPs were then recorded in animals injected with sivelestat. The sivelestat administration in the LPS and saline groups had no effect on nerve conduction evaluated by latency analysis (**Figures 6A, B**). Interestingly, the reduction in ET formation following the sivelestat injection in LPS-treated animals (LPS + sivelestat group) preserved the gastrocnemius MEP amplitude (significant from 80% to 100% MO) compared to the LPS group (26.13 ± 5.78 *vs*. 8.15 ± 5.89 mV, respectively, p<0.01), (**Figures 6C, D**).

## 4 Discussion

In this study, we showed for the first time that infiltrating neutrophils and microglia formed ETs in the spinal cord during endotoxemic shock. This production was associated with a reduction in motoneuronal excitability. Interestingly, the inhibition of ET production by β1-adrenergic blockers or a specific ET inhibitor (sivelestat) partially preserved motoneuronal function.

Neutrophils are peripheral immune cells known to display upregulation of their activities during severe inflammation, associated with infiltration of the CNS (14, 43) as shown in **Supplemental Figure 3**. This infiltration is due to an increase in leukocyte diapedeses or permeabilization of the blood–spinal cord barrier (BSCB), as shown in **Supplemental Figure 4**, facilitating the passage of these cells into the CNS (11). Neutrophils are also known for producing ETs (17) in various infectious or autoimmune pathologies and in different organs such as spinal cord tissue (21, 24, 44). However, the formation of ETs in the CNS during severe inflammation has never been described before and is not limited to neutrophils. As observed in this study, microglia can also produce ETs, as already observed *in vitro* (28) and *in vivo* (26, 27) in other pathologies impacting CNS homeostasis.

With dysregulation of ET production, adverse effects have been observed, such as vascular occlusion (18) and organ dysfunction (19, 20). These may be due to the intrinsic properties of ETs and the future of the cells that release them. Two types of ET have been observed: vital and suicidal. The fundamental differences are the nature of the inciting stimuli and the timing of the ET release. The formation of suicidal ETs has been primarily demonstrated in the context of chemical stimulation such as PMA (Phorbol 12-myristate 13-acetate) and requires hours (45). In contrast, the formation of vital ETs has been demonstrated with microbial molecular models, particularly after LPS administration, and is achieved much faster (45). Thus, the analysis of ET formation in this present study was restricted, to the vital form with the expression of specific ET cell markers on alive cells with intact membranes. We also analyzed the combination of markers such as H3 citr and MPO, considered representative of specific vital ETs formation (46). H3 citr is involved in chromatin decondensation, and MPO, a granular enzyme, is involved in nuclear decondensation. In addition to H3 citr and MPO for ET labeling, SYTOX, a DNA intercalant agent, and TFAM, a protein associated with mitochondrial DNA (40, 47), were used. While the four markers confirmed the production of ETs during severe inflammation for neutrophils and microglia, TFAM also suggested an initial production only a few hours post infection. Vital ETs are probably formed by nuclear DNA at the initial stage, and later by mitochondrial DNA (48).

Recently, it has been demonstrated that β1-adrenergic receptors could modulate NET production (34, 49). In addition, a reduction in the production of ETs in the lung has been reported in COVID-19 patients following β1-adrenergic blocker administration (34). However, β1-adrenergic blocker administration after a massive inflammation induced by an exogenous component, such as LPS, has never been evaluated in the CNS before. This ET modulation by ß1-adrenergic blockers (**Figures 2**, **3**) could be achieved either by a modulation of the number of immune cells infiltrated or by a modulation of the number of β1-adrenergic receptors expressed by these immune cells. The first hypothesis is not confirmed because the percentage of neutrophils or microglia among CD45 positive cells was similar between the LPS and LPS + atenolol groups (**Supplemental Figure 3**). Regarding the number of β1-adrenergic receptors present on immune cells, their expression at their surface has been debated. Neutrophils have long been shown to express β2-adrenergic receptors on their surface but no β1-adrenergic receptors (50), whereas recent studies have shown both receptors present on neutrophils (51). In this study, we confirmed that (1) neutrophils and microglia express β1-adrenergic receptors on their membrane surfaces, and (2) after systemic LPS administration, the number of membrane receptors is increased. Interestingly, the administration of ß1-adrenergic blockers did not significantly change the number of infiltrating neutrophils and microglia in the spinal cord (**Supplemental Figure 5**), whereas a reduction in ETs production was observed in the spinal cord tissue, (**Figures 2**, **3**) as well as a beneficial effect on neuromuscular function by preserving MEP amplitude (**Figure 4**). This last effect suggests a modulation of reactive oxygen species production in motoneurons. This oxidative stress is a common denominator in the pathology of neurodegenerative disorders.

To determine whether a direct link exists between the decrease in ET production after β1-blocker administration and the preservation of neuromuscular excitability, we injected a specific inhibitor of ET production. Sivelestat specifically inhibits elastase, which is required during the initial step of ET formation. Following the sivelestat injection, ET production was reduced (**Figure 5**), and MEP amplitude preservation was observed (**Figure 6**). This result suggests a deleterious role of ETs on neuromuscular function in our endotoxemic mice model, similar to what has been demonstrated in other pathologies, such as spinal cord injury, when inhibitors of ETs were administered. An improvement in motor function was observed following the administration of ET inhibitors such as Cl-amidine, which inhibits the enzyme peptidylarginine deiminase 4 (PAD4), involved in histone citrullination (24), based on the Basso, Beattie, and Bresnahan test score (BBB Score), or sivelestat, based on PEM amplitude and the BBB score (52). In sepsis, a study showed positive effects on neuromuscular function following the administration of ulinastatin, an inhibitor of serine proteases including elastase, which potentially allowed inhibiting the production of ETs, without having mentioned it (13).

### 4.1 Limits of the study and interest for humans

The concentration of β1-blockers was determined to limit significant effects on cardiovascular parameters such as heart rate and arterial pressure in our model (heart rate: -8.7 ± 1.8% of baseline value and mean arterial pressure: -12.5 ± 4.8% of baseline value at 6 hours post LPS injection and 10 minutes post atenolol administration). Moreover, atenolol has a good β1-adrenergic receptor specificity and a sufficient half-life for our protocol (53).

Interestingly, gastrocnemius MEPs (latency and amplitude) can at least partly reflect the state of the motor neurons, the nerve conduction, the neuromuscular junction, and the state of muscular contractility. LPS model being characterized by a systemic inflammation, we cannot rule out that the observed functional effect (MEP amplitude reduction) was not also partly due to muscle inflammation through, for example, ET production by neutrophils and monocytes or monocyte-derived macrophages that infiltrate the muscles.

### 4.2 Conclusion

In conclusion, our results show that LPS increased neutrophil infiltration in the spinal cord, activated microglia cells, led to ET formation, and decreased neuromuscular function. Moreover, the results showed a beneficial effect of β1-adrenergic blocker administration on ET formation and neuromuscular function evaluated by MEP amplitude. The link between ETs and neuromuscular function was confirmed following the administration of a specific inhibitor of ETs. Thus, the modulation of ET formation during inflammatory-related diseases could have beneficial effects on patients to limit neuromuscular function disagreements. It could also be beneficial for other neuroinflammatory pathologies associated with motor function disorders, such as spinal cord injuries.

## Data availability statement

The original contributions presented in the study are included in the article/**Supplementary Material**. Further inquiries can be directed to the corresponding author.

## Ethics statement

The animal studies were approved by Ethics committee of the University of Versailles Saint- Quentin-en-Yvelines (CEEA-47; APAFIS #38573-2022082610387745 v4). The studies were conducted in accordance with the local legislation and institutional requirements. Written informed consent was not required from the owners for the participation of their animals in the study in accordance with local legislation and institutional requirements.

## Author contributions

CB: conceptualization, investigation, formal analysis, visualization, project administration, writing—original draft and review and editing. PM-F: conceptualization, methodology, investigation and writing—review and editing. LE: investigation, formal analysis, review and editing. LA: investigation, formal analysis, review and editing. AG: investigation, review and editing. SV: conceptualization, methodology, supervision, project administration, funding acquisition and writing—review and editing. AM: conceptualization, methodology, supervision, project administration, funding acquisition and writing—review and editing. All authors contributed to the article and approved the submitted version.

## Supplementary material

Supplementary Figure 1 Experimental protocol. A single injection of lipopolysaccharides (LPS) was used to create our preclinical endotoxemic shock model. Mice were divided in different groups: LPS group **(A)**, LPS + atenolol (atenolol was injected 6 hours after LPS injection) **(A)**, LPS + sivelestat group (sivelestat injected at 4 hours (10 mg/kg), 8 hours (20 mg/kg), and 12 hours (20 mg/kg) after LPS injection) **(B)**.Controls groups were also used for each condition: saline group (NaCl 0.9%) **(A)**, atenolol group (atenolol was injected 6 hours after the injection saline) **(A)**, and sivelestat group (3 of sivelestat injections 4, 8, and 12 hours after saline injection) **(B)**.

Supplementary Figure 2 Gating strategy for neutrophils and microglia. Gating strategy used to differentiate CD11b^+ and^ CD45^int^ microglia and CD11b^+^, CD45^high and^ Ly6G^+^ neutrophils for a representative animal of saline, LPS, and LPS + atenolol groups.

Supplementary Figure 3 Frequency of neutrophils and microglia among living cells in spinal cord. Flow cytometry analysis of the frequency of neutrophils among living cells, in the saline, atenolol, LPS, and LPS + atenolol **(A)** groups. Flow cytometry analysis of the frequency of microglia among living cells, in saline, atenolol, LPS and LPS + atenolol groups **(B)** groups. Representative picture of entire spinal cord transversal section (low thoracic to high lumbar segment), labeled with Ly6G (neutrophil marker, green), for saline and LPS groups **(C)**, counterstained with DAPI (blue). The grey matter is delimited with white dashed line. *** p<0.001.

Supplementary Figure 4 LPS administration promoted blood–spinal cord barrier disruption. Quantification of Evans blue (EB) leakage in cervical spinal cord **(A)**. Representative fluorescence image of FITC (green) in blood vessels in cervical ventral spinal cord for saline and LPS groups **(B)**. White arrow show an increase in FITC leakage from blood vessel in the LPS group. *** p<0.001.

Supplementary Figure 5 LPS increased the expression of β1-adrenergic receptors in neutrophils and microglia. Fluorescence intensity for a representative animal of each group for blood neutrophils **(A)**, neutrophils in spinal cord **(B)**, and microglia **(C)**. Quantification of mean fluorescence intensity (MFI) of β1-adrenergic receptors (ADRB1) on blood neutrophils **(D)**, spinal cord neutrophils, **(E)** and microglia **(F)**. *** p<0.001.
