# Supplementary figures and images for: ß1-adrenergic blockers preserve neuromuscular function by inhibiting the production of extracellular traps during systemic inflammation in mice
Source: Front Immunol. 2023 Sep 22;14:1228374. doi: 10.3389/fimmu.2023.1228374 (PMC10556451; doi:10.3389/fimmu.2023.1228374)

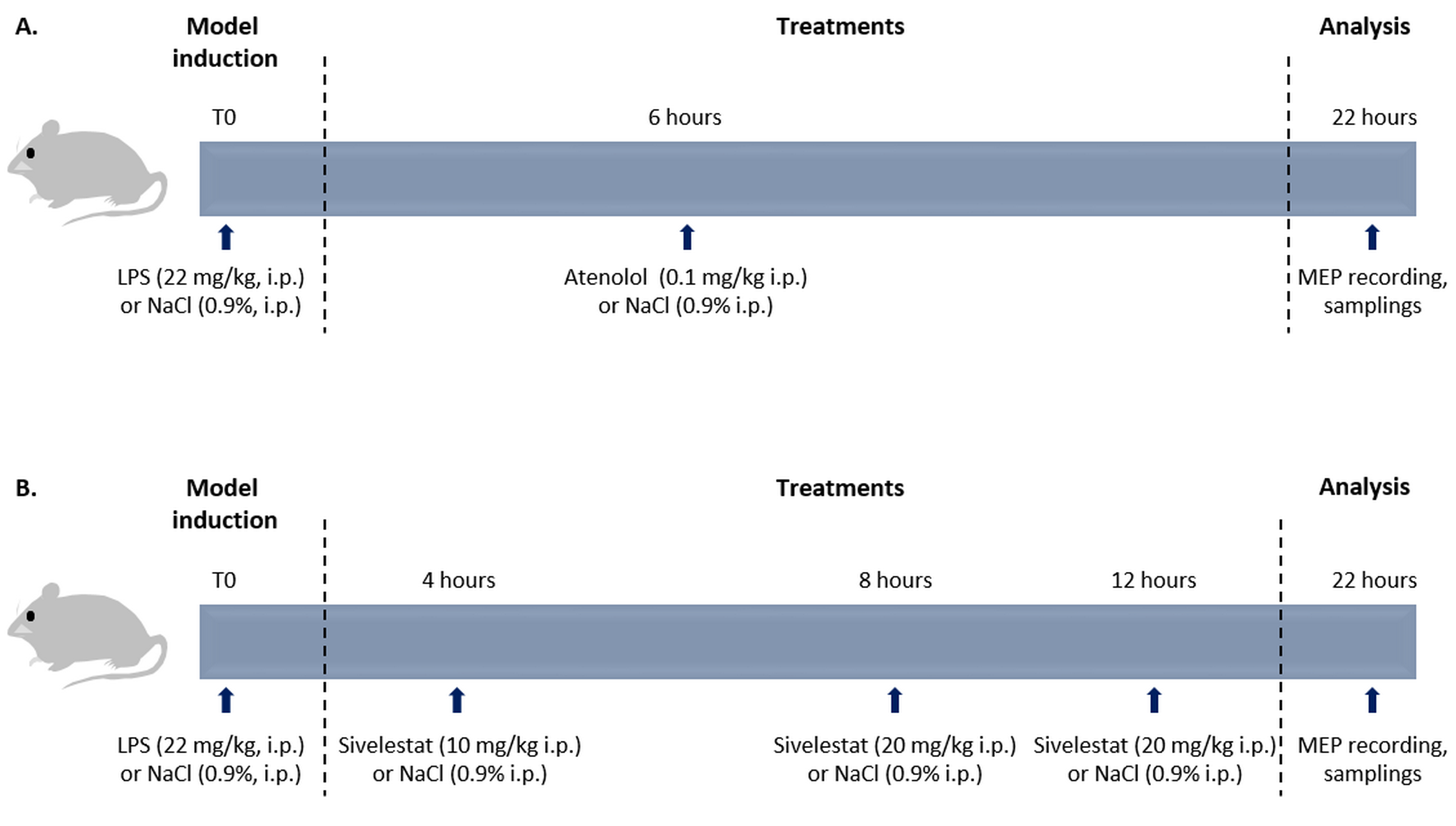

Supplement: Supplementary Figure 1 — Experimental protocol. A single injection of lipopolysaccharides (LPS) was used to create our preclinical endotoxemic shock model. Mice were divided in different groups: LPS group (A), LPS + atenolol (atenolol was injected 6 hours after LPS injection) (A), LPS + sivelestat group (sivelestat injected at 4 hours (10 mg/kg), 8 hours (20 mg/kg), and 12 hours (20 mg/kg) after LPS injection) (B).Controls groups were also used for each condition: saline group (NaCl 0.9%) (A), atenolol group (atenolol was injected 6 hours after the injection saline) (A), and sivelestat group (3 of sivelestat injections 4, 8, and 12 hours after saline injection) (B). [file Image_1.tif]

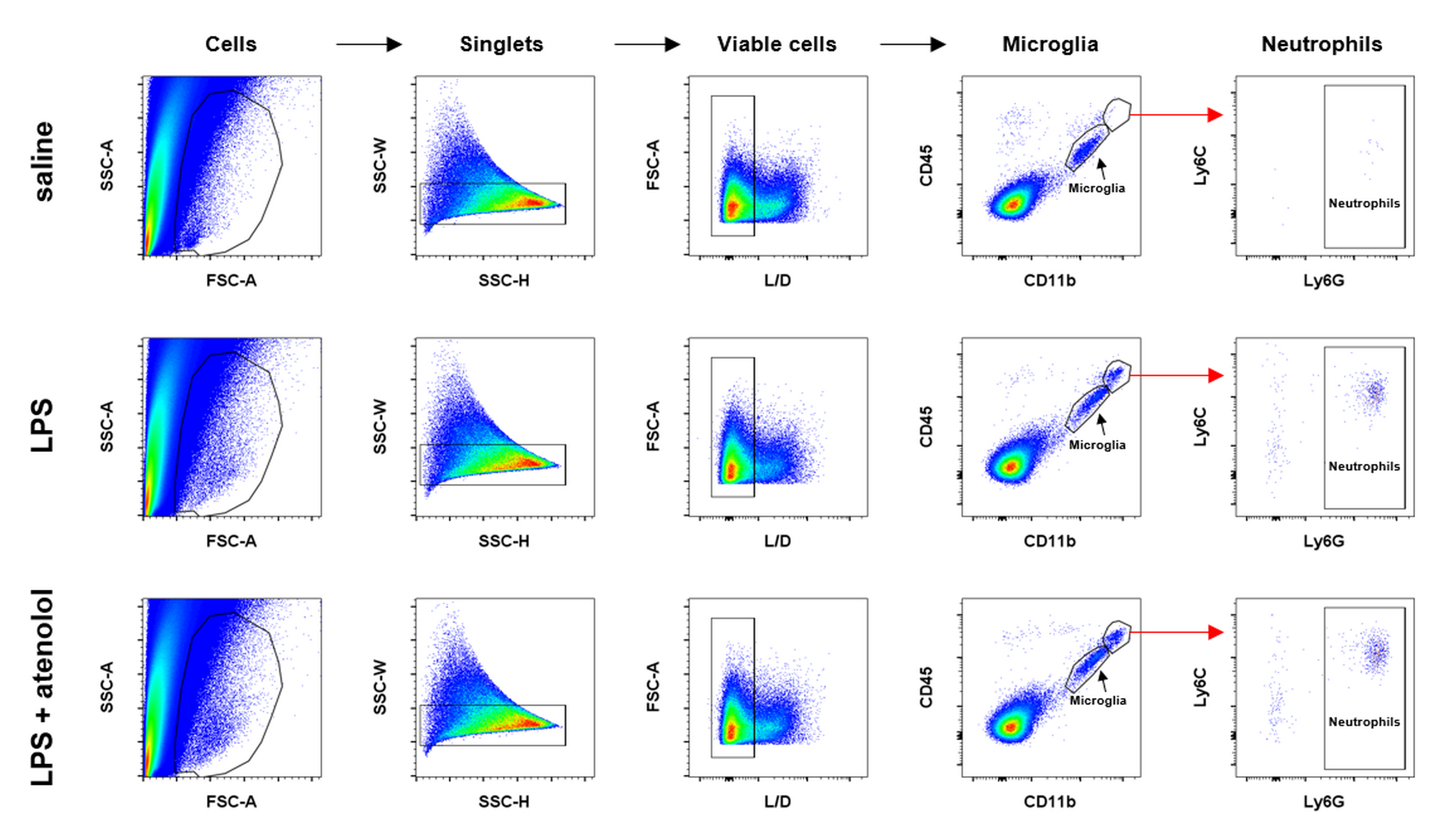

Supplement: Supplementary Figure 2 — Gating strategy for neutrophils and microglia. Gating strategy used to differentiate CD11b+ and CD45int microglia and CD11b+, CD45high and Ly6G+ neutrophils for a representative animal of saline, LPS, and LPS + atenolol groups. [file Image_2.tif]

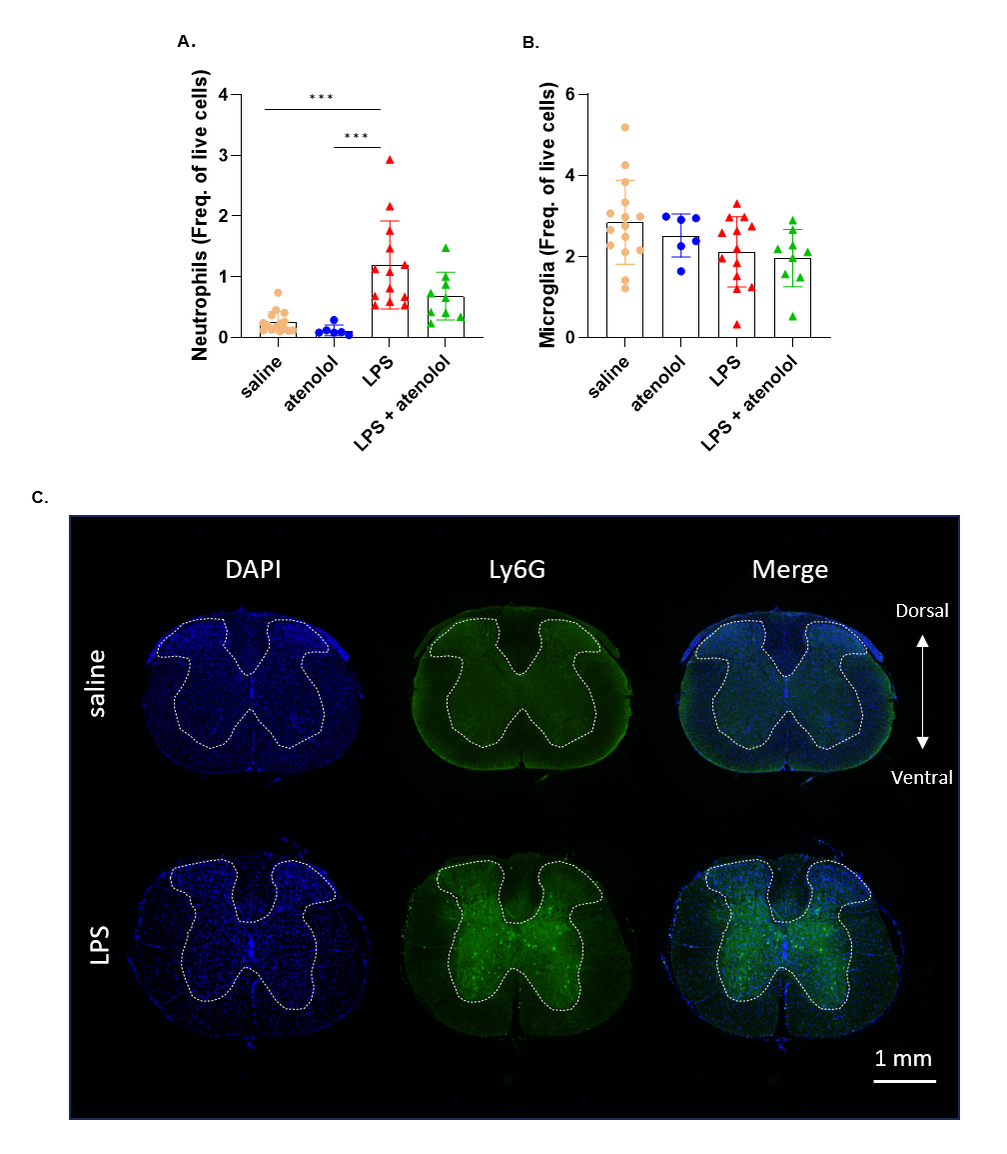

Supplement: Supplementary Figure 3 — Frequency of neutrophils and microglia among living cells in spinal cord. Flow cytometry analysis of the frequency of neutrophils among living cells, in the saline, atenolol, LPS, and LPS + atenolol (A) groups. Flow cytometry analysis of the frequency of microglia among living cells, in saline, atenolol, LPS and LPS + atenolol groups (B) groups. Representative picture of entire spinal cord transversal section (low thoracic to high lumbar segment), labeled with Ly6G (neutrophil marker, green), for saline and LPS groups (C), counterstained with DAPI (blue). The grey matter is delimited with white dashed line. *** p<0.001. [file Image_3.tif]

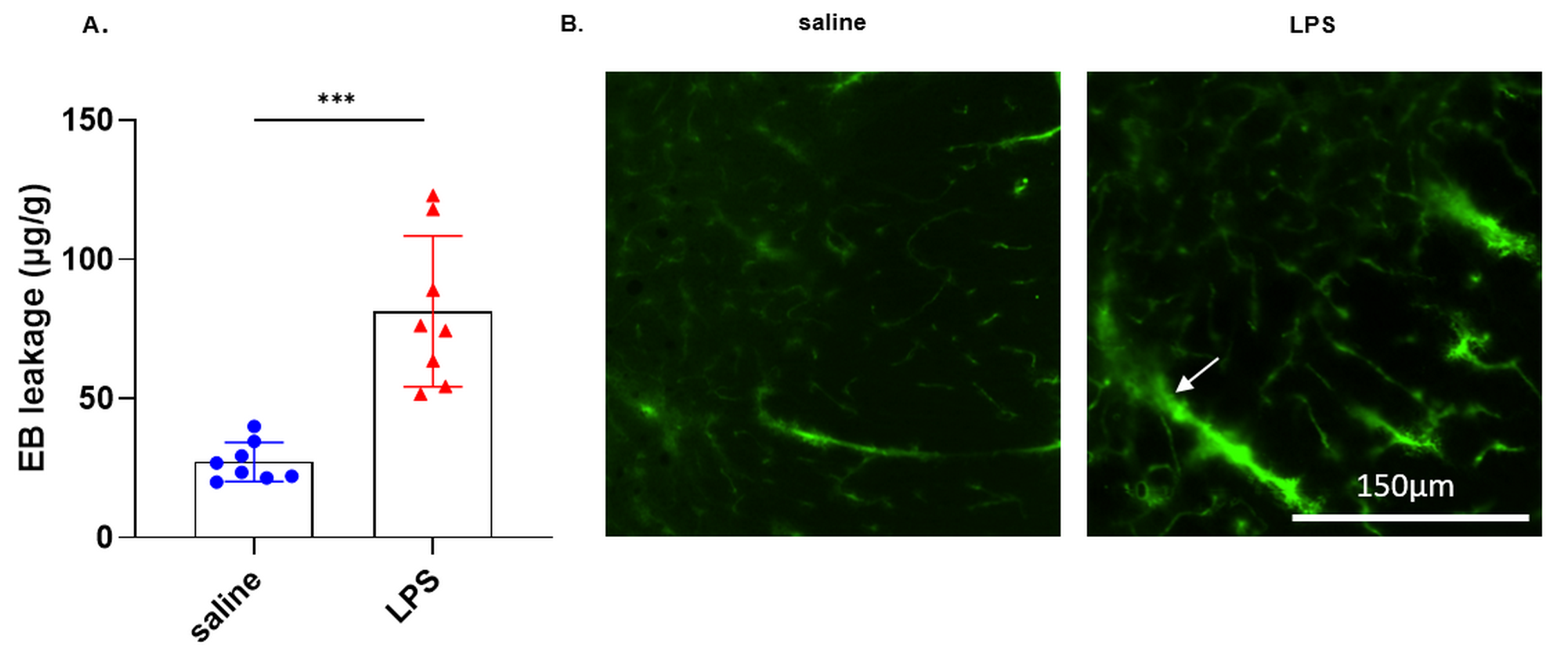

Supplement: Supplementary Figure 4 — LPS administration promoted blood–spinal cord barrier disruption. Quantification of Evans blue (EB) leakage in cervical spinal cord (A). Representative fluorescence image of FITC (green) in blood vessels in cervical ventral spinal cord for saline and LPS groups (B). White arrow show an increase in FITC leakage from blood vessel in the LPS group. *** p<0.001. [file Image_4.tif]

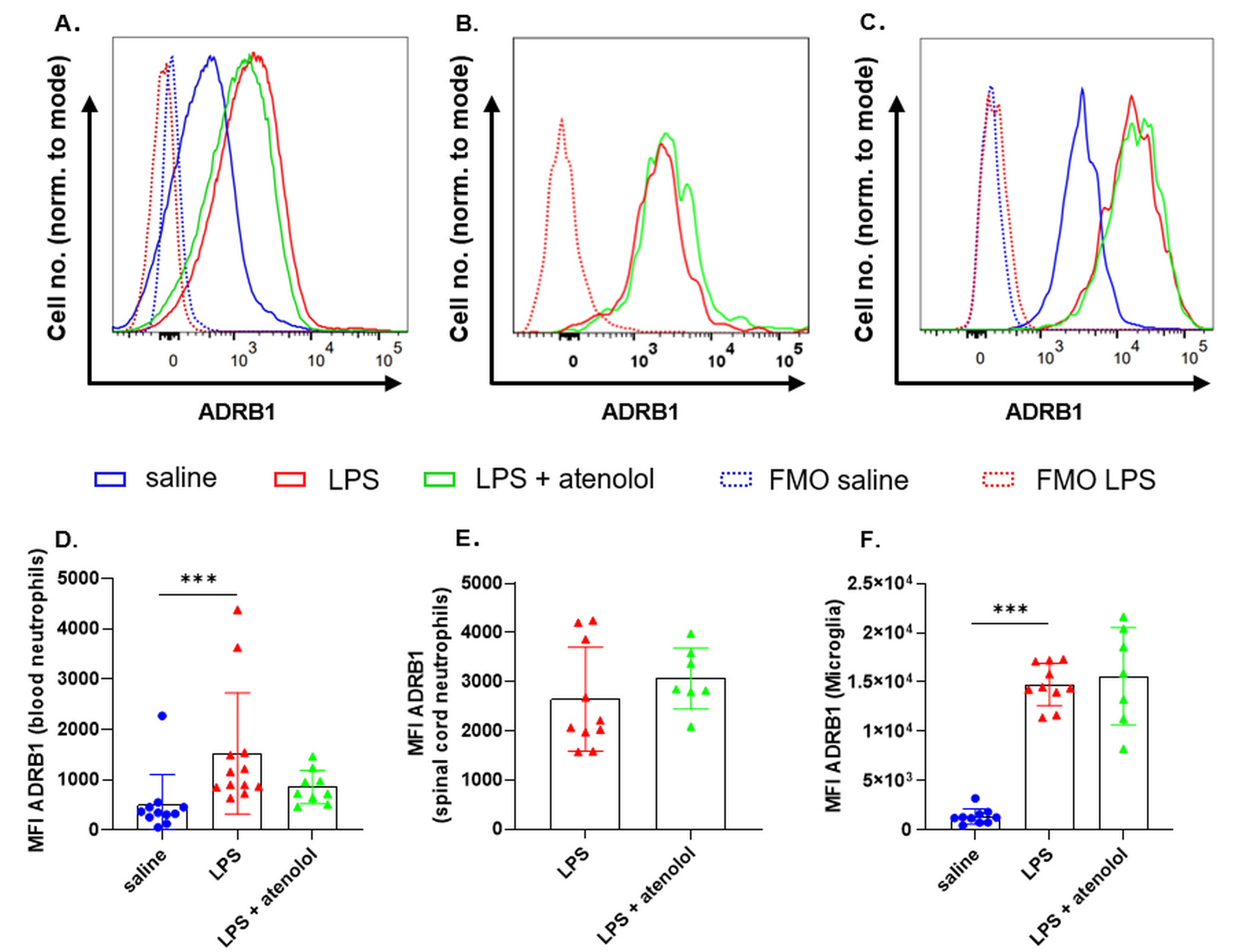

Supplement: Supplementary Figure 5 — LPS increased the expression of β1-adrenergic receptors in neutrophils and microglia. Fluorescence intensity for a representative animal of each group for blood neutrophils (A), neutrophils in spinal cord (B), and microglia (C). Quantification of mean fluorescence intensity (MFI) of β1-adrenergic receptors (ADRB1) on blood neutrophils (D), spinal cord neutrophils, (E) and microglia (F). *** p<0.001. [file Image_5.tif]

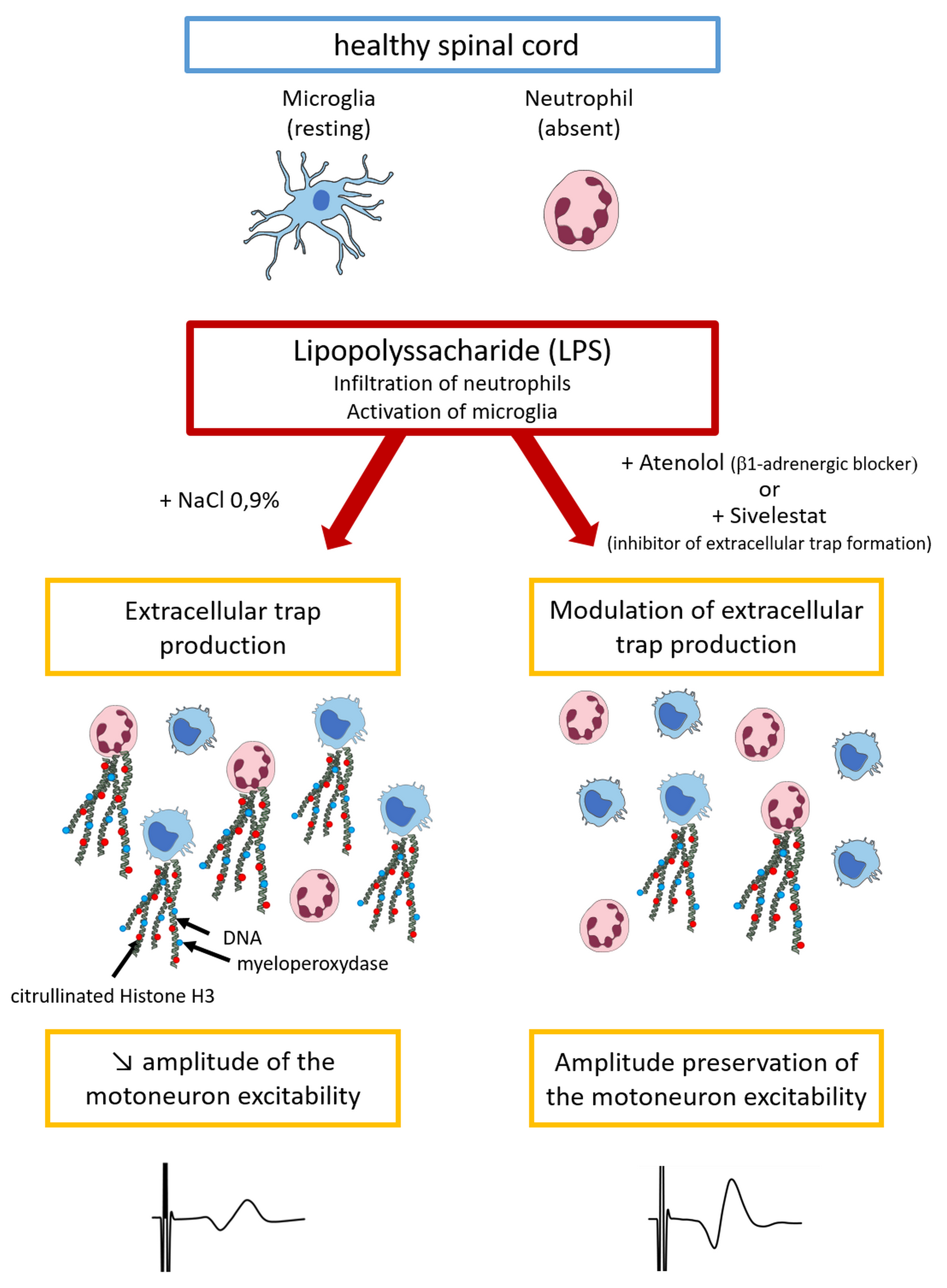

Supplement: Supplementary file 6 [file Image_6.tif]
